# Myofibroblastoma of the Breast: A Case Report Highlighting the Importance of Accurate Diagnosis and Multidisciplinary Management

**DOI:** 10.7759/cureus.85418

**Published:** 2025-06-05

**Authors:** Jose C Ramos, Anjeza Chukus

**Affiliations:** 1 Diagnostic Radiology, Hospital Corporation of America (HCA) Florida Aventura Hospital, Aventura, USA; 2 Neuroradiology and Breast Imaging, Hospital Corporation of America (HCA) Florida Aventura Hospital, Aventura, USA

**Keywords:** core needle biopsy, hypoechoic breast mass, immunohistochemical markers, myofibroblastoma, spindle cell tumor, well-circumscribed mass

## Abstract

Myofibroblastoma (MFB) of the breast is a rare benign mesenchymal tumor originating from mammary stromal cells, posing diagnostic challenges due to its diverse presentation. It typically affects older individuals, often postmenopausal women and men aged 40-87 years, presenting as a solitary, painless, well-defined, mobile mass that grows slowly. On mammography, MFB typically manifests as a well-circumscribed, non-calcified, round or oval mass, often of equal or high density, though margins can sometimes be obscured or indistinct, and rare coarse calcifications have been reported. Ultrasound findings are variable but often demonstrate a solid, irregular, non-circumscribed mass with varying posterior acoustic features and vascularity. Diagnosing MFB requires core needle biopsy and immunohistochemistry, as imaging findings are non-specific. However, MFB encompasses a broad morphologic spectrum beyond the classic spindle cell type and sometimes demonstrate mixed variants that can exhibit unusual or alarming features that make diagnosis challenging. A key feature is the presence of interspersed thick, hyalinized, and eosinophilic collagen bundles. Immunohistochemically, MFB characteristically shows strong positivity for CD34 and desmin. Treatment involves surgical excision, which is curative, and the prognosis is excellent with an extremely low risk of recurrence and no metastatic potential. Accurate differentiation from malignant mimics, particularly invasive lobular carcinoma and metaplastic carcinoma, is crucial. This case report describes a 41-year-old female who was found to have a suspicious right breast mass on a diagnostic ultrasound, later confirmed as MFB through pathology. Given the rarity of MFB, this case underscores the importance of accurate diagnosis and a multidisciplinary approach involving radiology, pathology, and surgery for appropriate management.

## Introduction

Myofibroblastoma (MFB) of the breast is a rare benign mesenchymal tumor that can be challenging to diagnose due to its varied morphological patterns [1-6]. Mesenchymal tumors arise from connective tissues, which include muscle, fat, fibrous tissue, blood vessels, and lymphatics, and can occur in various parts of the body, such as the skin, soft tissues, visceral organs, and the gastrointestinal (GI) tract [1-6]. First described in 1987, it is defined by spindle cell proliferation with myofibroblastic differentiation [1-8]. Despite its rarity, recognizing MFB is critical for distinguishing it from other benign and malignant breast lesions [2,4-6,8,9-14].

MFB affects both sexes and occurs across a wide age range, typically between 25 and 87 years, without preference for any race or ethnicity [3,4,6,8,10]. It most commonly arises in the breast parenchyma but may also develop in extramammary locations along the embryonic milk line [1-3,6,9,12]. Clinically, it presents as a painless, mobile, well-defined mass [1-5,9,13]. Its histological variants, including collagenized, cellular, lipomatous, myxoid, and epithelioid patterns, can complicate diagnosis [1,2,4,8,13].

Imaging findings are non-specific, often mimicking fibroadenomas or other benign masses [1,2,5,6,8,9,12,13]. On ultrasound, MFB appears as a well-circumscribed, hypoechoic mass, while mammography typically shows a round or oval dense lesion without calcifications [1-3,5-9,12,14]. A definitive diagnosis requires needle biopsy and immunohistochemical analysis [1-9,14].

Treatment involves local surgical excision with clear margins, which is curative [1-3,5-11,13]. Recurrence is rare, and no cases of malignant transformation have been reported [1,2,5,6,9,10,13]. Radiation or systemic therapies are unnecessary, though follow-up for at least 24 months is recommended to ensure complete resolution [2,5,6,9,13,14].

Here, we present a case of myofibroblastoma of the breast, detailing its imaging and histopathological features. This report adds to the growing body of literature, emphasizing its diagnostic challenges and management strategies to improve recognition of this rare entity.

## Case presentation

**Figure 1:**
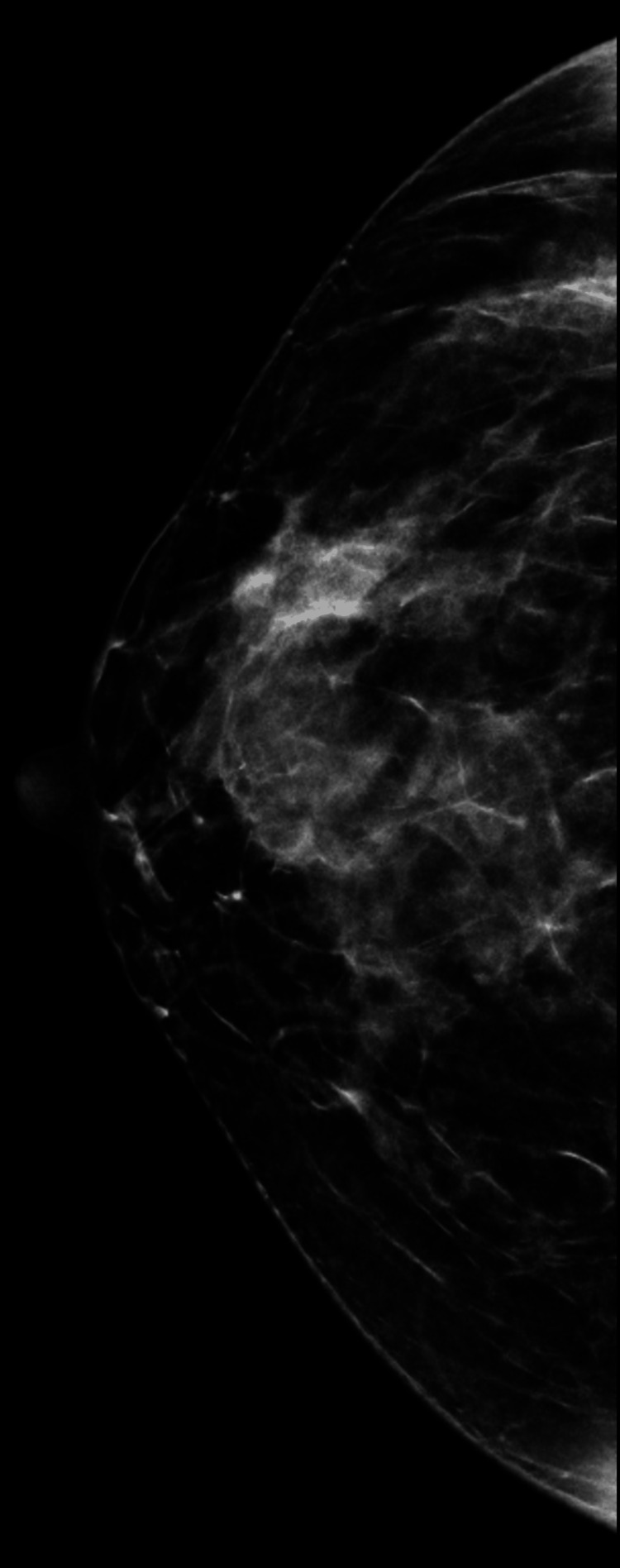
Right breast mammogram craniocaudal (CC) view. Heterogeneously dense right breast which may obscure small masses.

**Figure 2:**
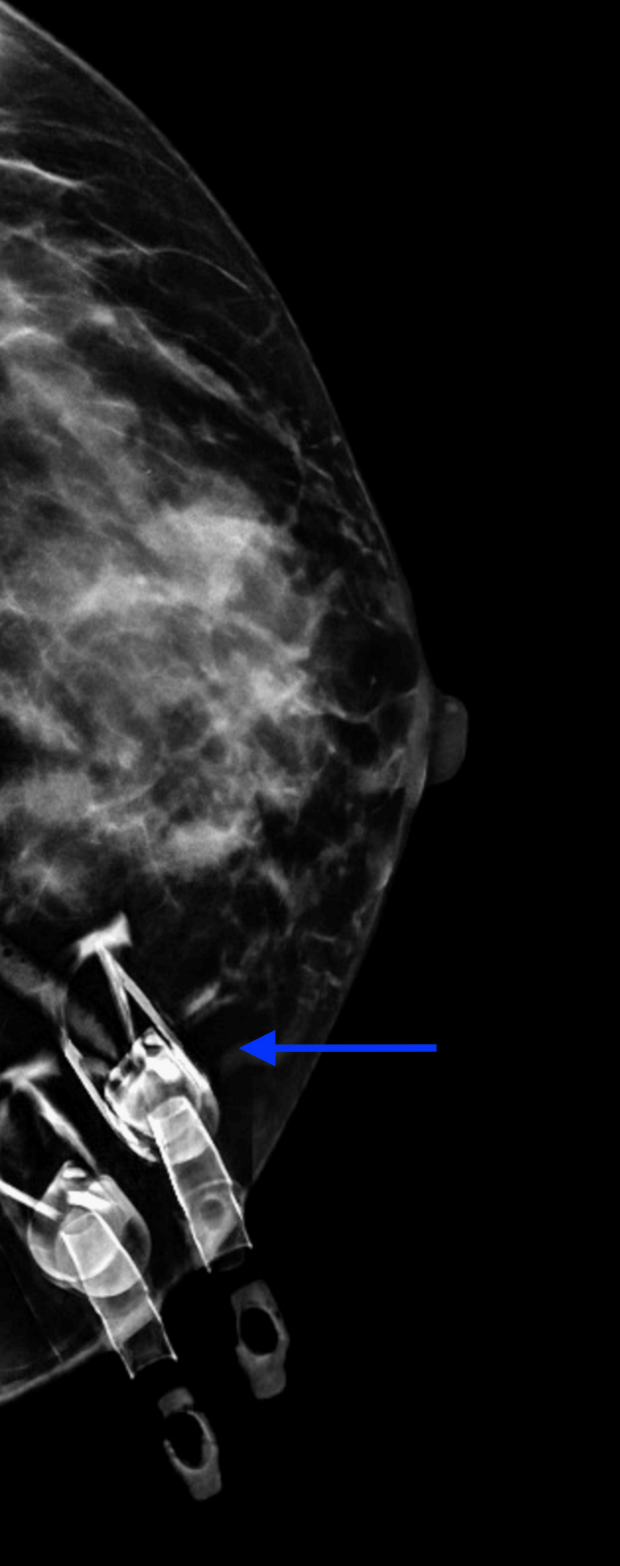
Left breast mammogram craniocaudal (CC) view. Arrow demonstrating an overlying catheter clamps limiting evaluation of the left inner breast.

**Figure 3:**
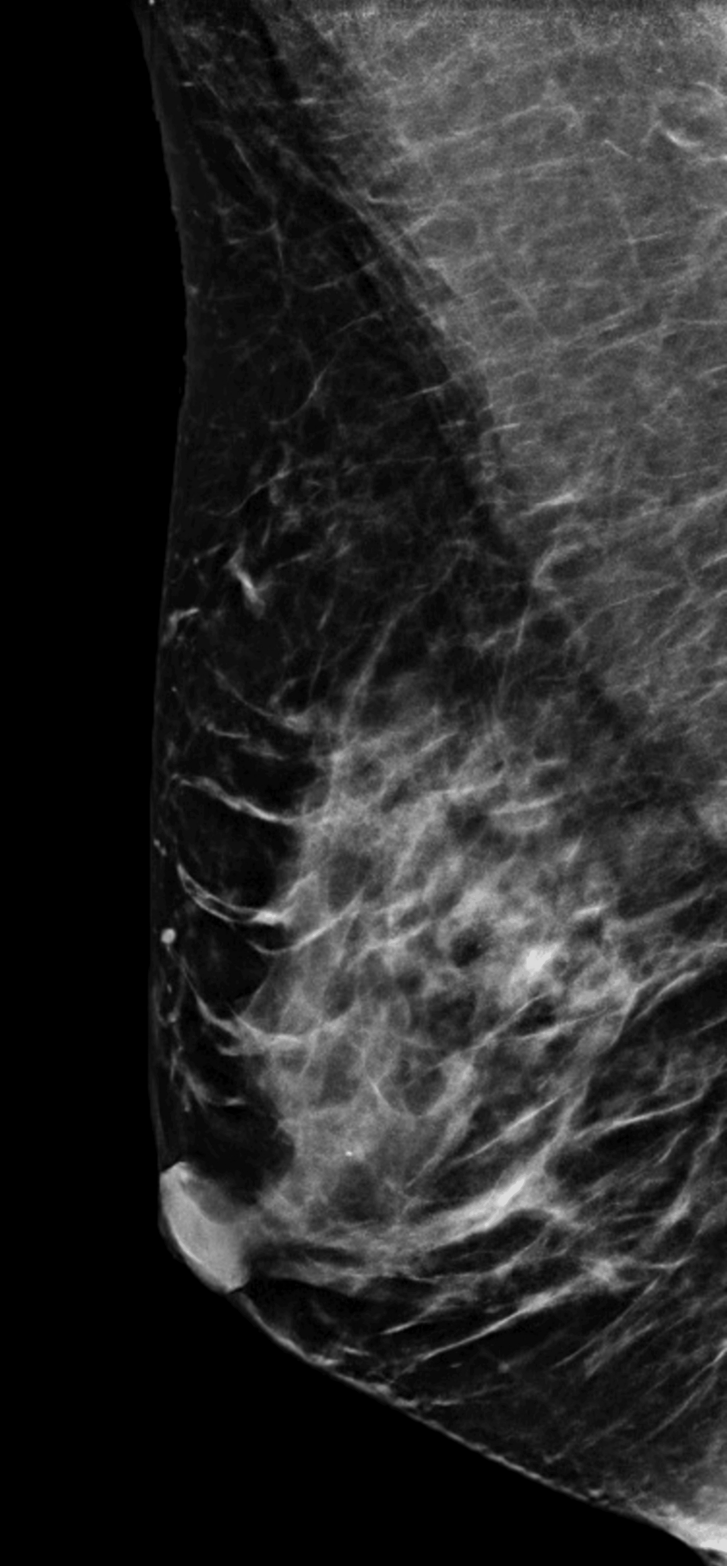
Right breast mammogram mediolateral oblique (MLO) view. Heterogeneously dense right breast which may obscure small masses.

**Figure 4:**
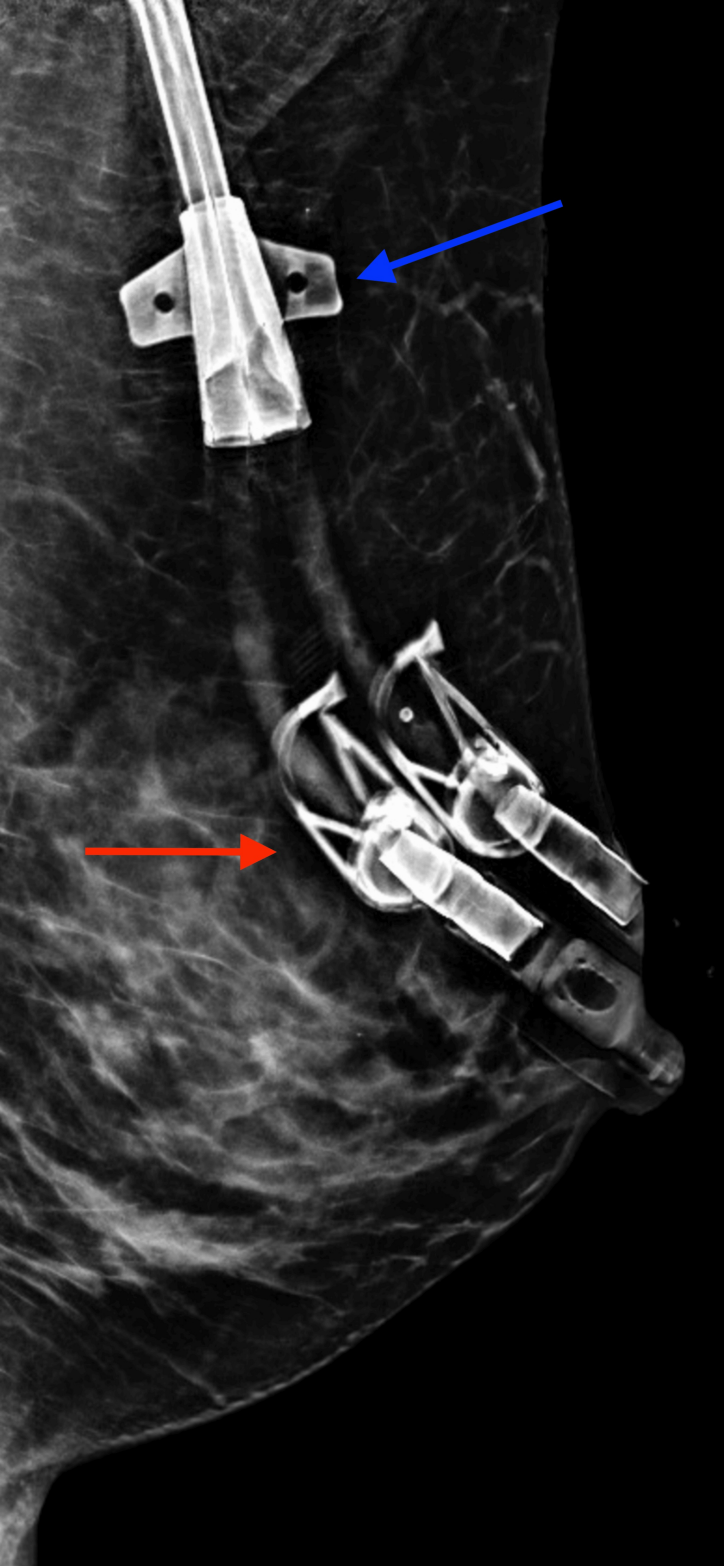
Left breast mammogram mediolateral oblique (MLO) view. Blue arrow demonstrating overlying catheter limiting evaluation of the left upper breast and axillary tail. Red arrow demonstrating overlying catheter clamps limiting evaluation of the left upper breast.

We present a case of a 41-year-old asymptomatic Hispanic female with no personal or family history of breast cancer that presented for her first bilateral screening mammogram (Figures 1-4).Patient was found to have bilateral heterogeneously dense breasts which can obscure small masses. Additionally, the patient was given a Breast Imaging Reporting and Data System (BIRADS) category 0 due to suboptimal imaging of the left upper breast from overlying catheter and occlusion clamps limiting visualization of the underlying breast. A subsequent bilateral diagnostic ultrasound was recommended. Patient underwent bilateral breast ultrasound one and a half months later. Among multiple benign findings, including bilateral benign-appearing cysts, a suspicious 1.19 cm right breast mass in the 10 o’clock position, approximately 4 cm from the nipple, was visualized.

**Figure 5:**
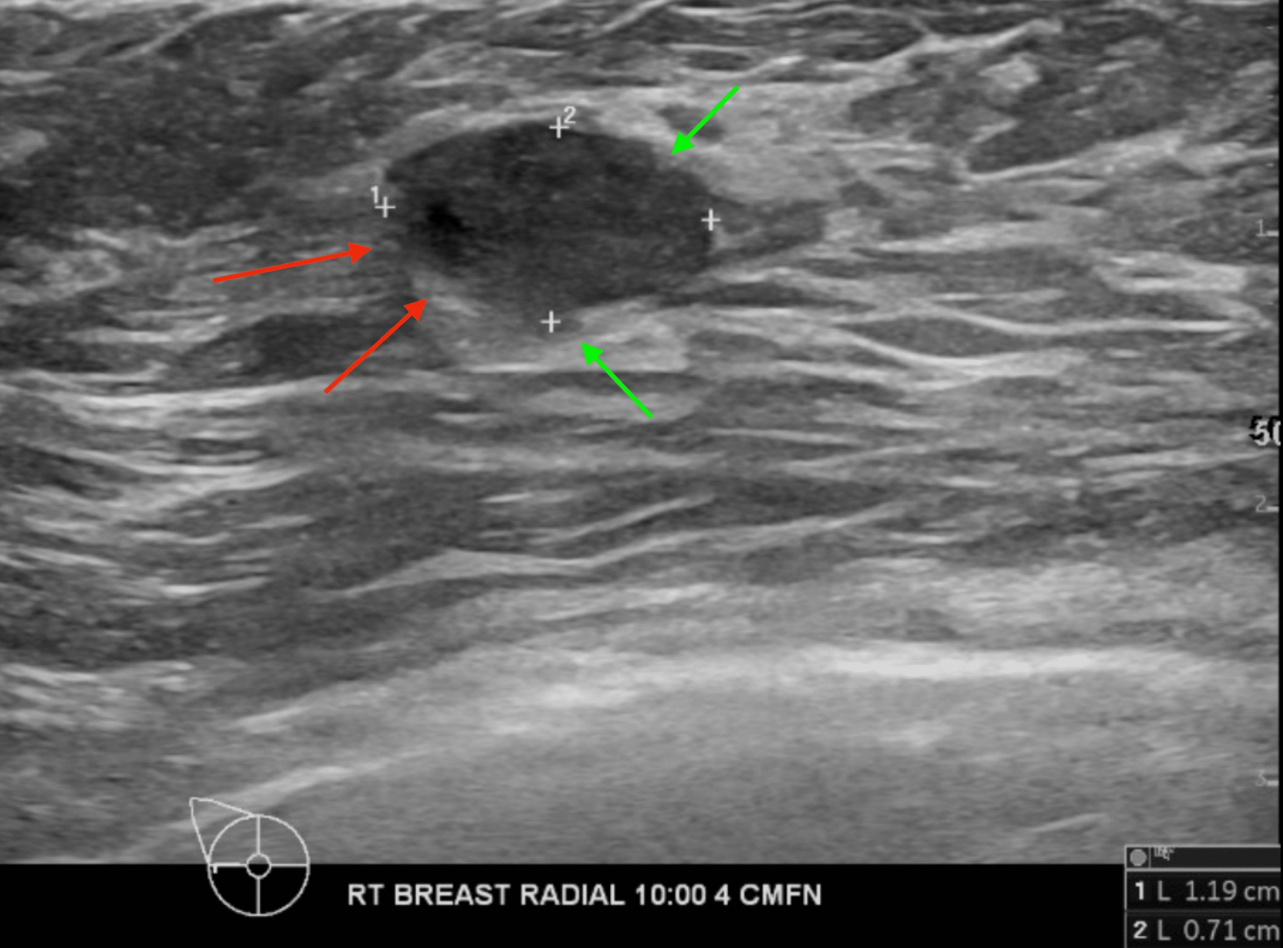
Right breast ultrasound 10 o'clock, 4 cm from the nipple demonstrating a 1.19 x 0.71 cm irregular hypoechoic mass with indistinct and angular borders. Red arrows demonstrating the indistinct margins of the right breast mass. Green arrows demonstrating microlobulated margins of the right breast mass.

**Figure 6:**
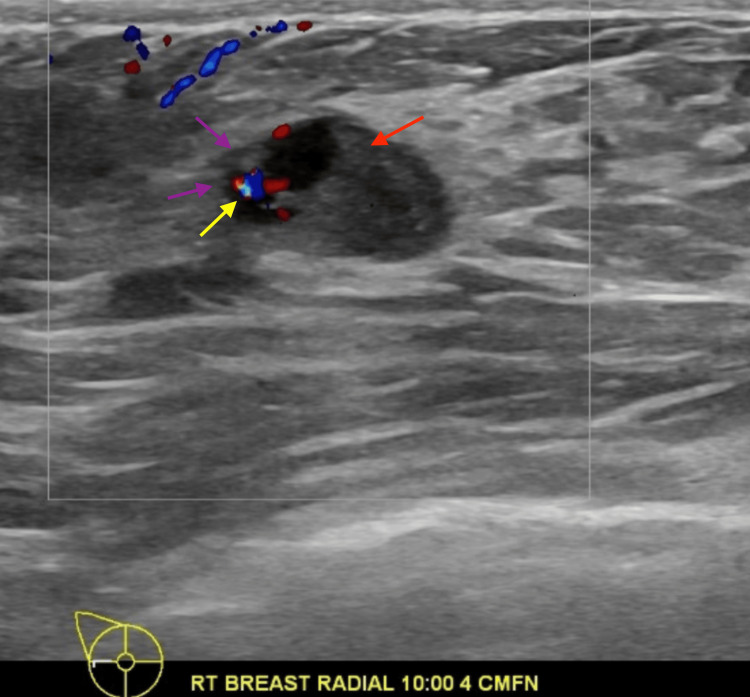
Right breast ultrasound 10 o'clock, 4 cm from the nipple demonstrating a complex cystic and solid mass with internal vascularity and indistinct borders. Yellow arrow demonstrating internal color Doppler flow. Purple arrow demonstrating indistinct borders. Red arrow demonstrating solid component

**Figure 7:**
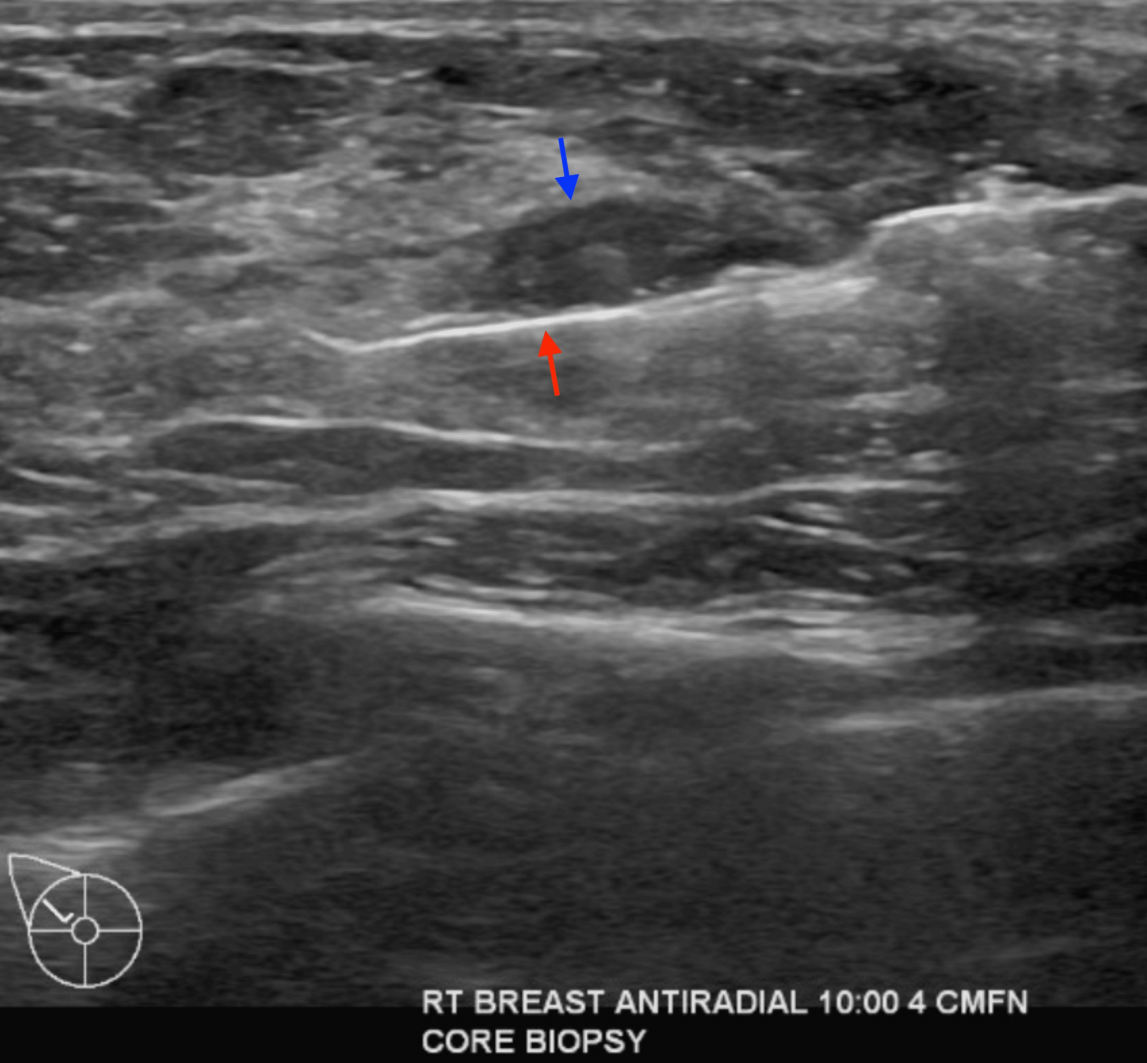
Right breast ultrasound-guided core needle biopsy of a 10 o'clock mass, 4 cm from the nipple. Blue arrow demonstrating right breast mass. Red arrow demonstrating biopsy needle within the mass.

**Figure 8:**
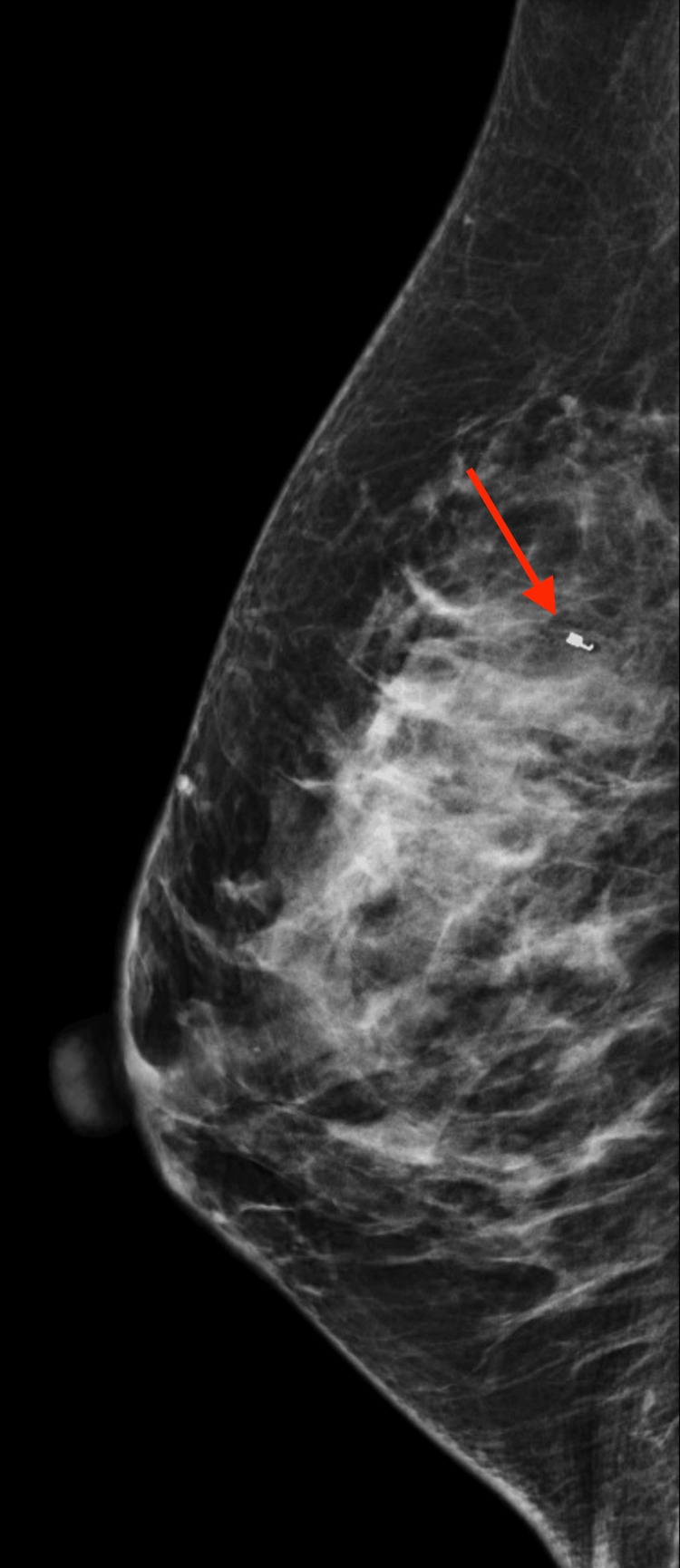
Right breast mammogram mediolateral (ML) view. The breast is heterogeneously dense, which obscures the biopsied mass. Arrow demonstrating adequate placements of a post-biopsy coil-shaped clip in the right upper breast.

**Figure 9:**
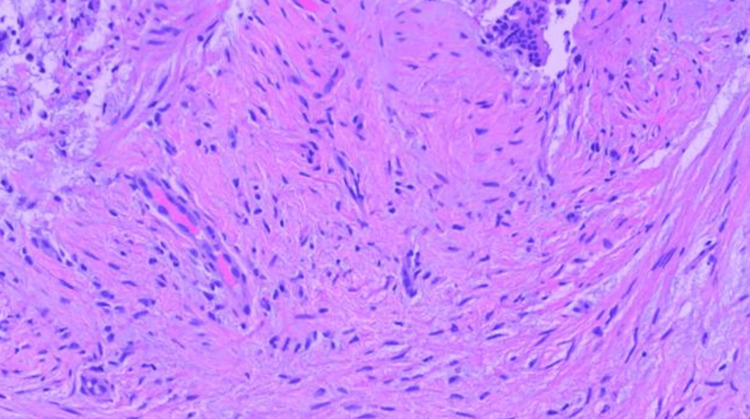
High power magnification demonstrating spindle cell proliferation.

**Figure 10:**
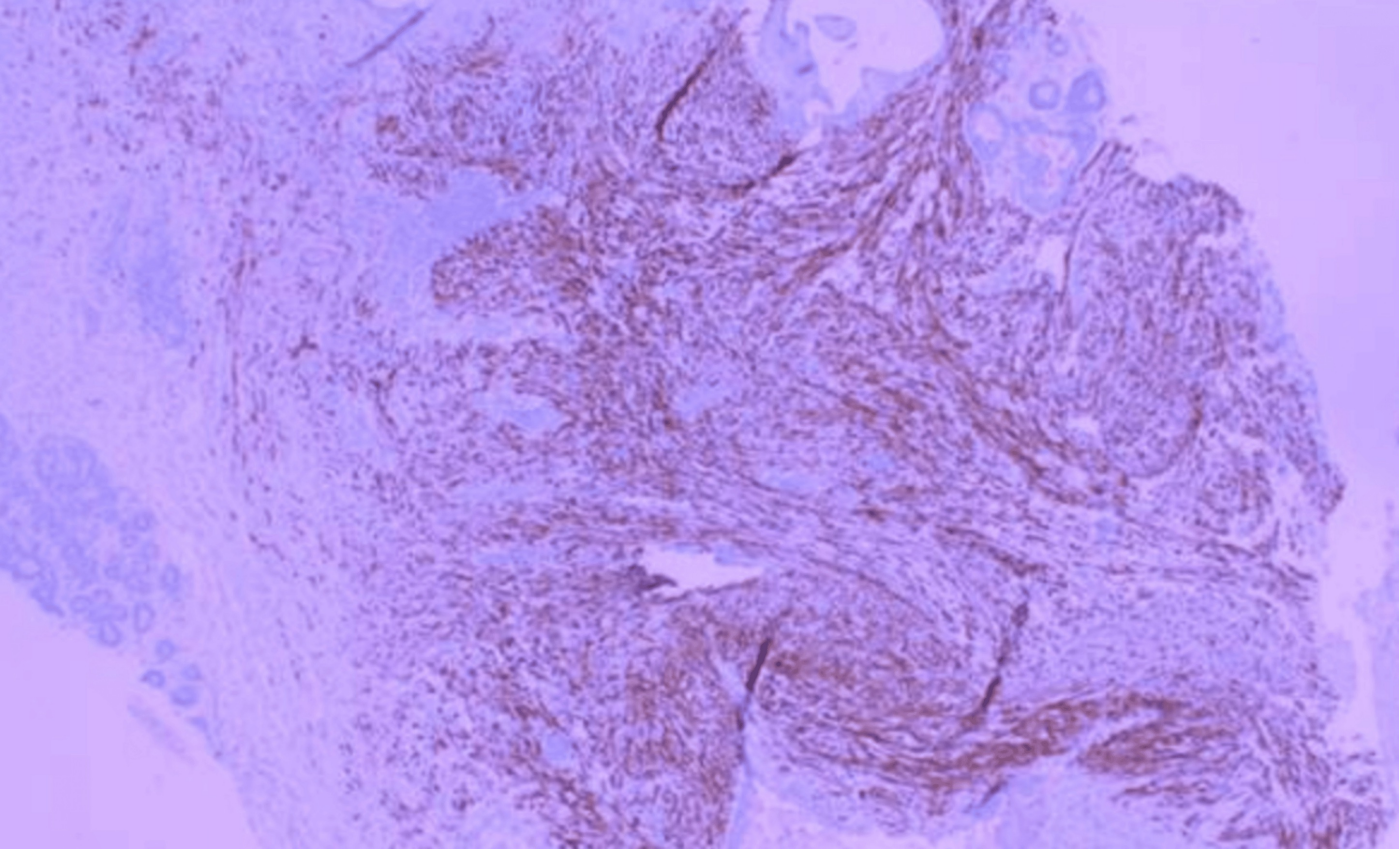
High power magnification demostrating desmin positive staining.

**Figure 11:**
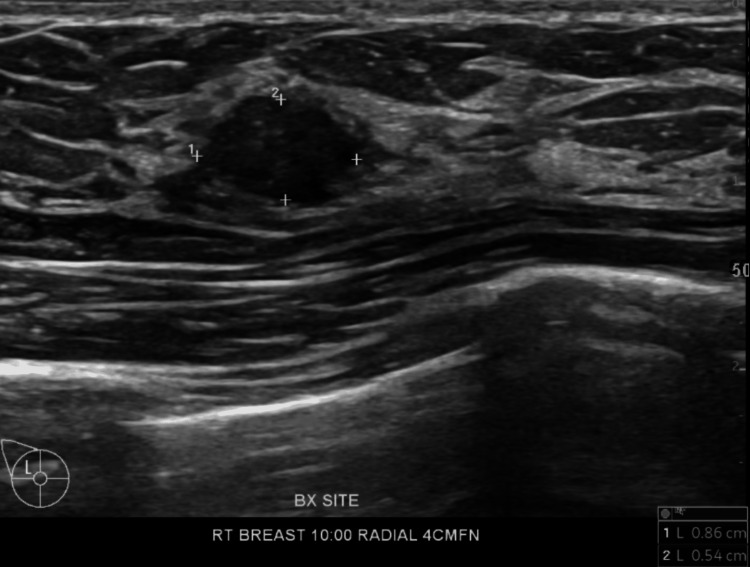
Right breast ultrasound six months post-core needle biopsy of a 10 o'clock mass 4 cm from the nipple measuring 0.86 x 0.54 cm

The mass showed oval shape with parallel orientation, with complex cystic and solid internal echogenicity, indistinct and microlobulated margins, and internal vascularity (Figures 5, 6). These findings were highly suspicious for malignancy, and hence, a BIRADS 4 was given with recommendations to undergo an ultrasound-guided core needle biopsy. On the day of the procedure, a repeat targeted right breast ultrasound of the 10 o’clock position redemonstrated an irregular mass with indistinct and microlobulated margins, keeping with previously visualized malignant characteristics and confirming the target area of concern. A biopsy was performed, acquiring three samples using a 14-gauge Celero biopsy core needle (Marlborough, MA: Hologic, Inc.) under ultrasound guidance (Figure 7). A post-biopsy right breast mammogram confirmed clip marker placement in the mass (Figure 8). Tissue cores were sent to pathology later, yielding benign breast tissue with stromal spindle cell proliferation, positive for calponin and desmin stains, most consistent with myofibroblastoma (Figures 9, 10). The patient was recommended for surgical consultation with a breast surgeon to discuss appropriate management. The patient opted against further procedures due to unknown circumstances. A six-month follow-up of the mass was recommended to assess stability. The follow-up showed stable characteristics of the right breast 10 o’clock mass (Figure 11).

## Discussion

Myofibroblastoma (MFB) of the breast is a rare, benign mesenchymal tumor that presents diagnostic challenges due to its diverse clinical and histological characteristics [1,2,4-9,11,13]. This study highlights the clinical relevance of MFB and the critical considerations for its accurate diagnosis and management [1-4,6,8,9,11-13].

MFB is an uncommon tumor, most often reported in postmenopausal women and older men, typically between the ages of 25 and 87 years [1-5,8,9,11,13]. While some evidence suggests a slight male predisposition, the tumor can occur in both sexes, with no racial predilection [1-4,6,13]. The limited epidemiological data underscore the rarity of MFB and its underrepresentation in clinical practice [1,2,4,6,9,11,13].

Clinically, MFB often presents as a painless, firm, and freely mobile mass that may grow slowly over time [1-3,5,8,9,12]. Although usually solitary, variations in presentation exist, with some cases associated with gynecomastia [1-3,6,8,12,13]. Tumor size can range from a few millimeters to over 10 cm [1,4,6,7-9,13]. The appearance of MFB on imaging studies such as mammography and ultrasound is often non-specific [1-3,5,6,8,9-11,13,14] They typically appear as either well-circumscribed or obscured dense masses on mammography [2,3,5,6,8,10,13,14]. Also, they can show indistinct borders with variable echogenicity on ultrasound, just as in the previously described case [1-3,5,6,8,9,12,13]. These findings can mimic benign and malignant masses, like fibroadenomas and malignant tumors highlighting the need for tissue-based diagnosis [1,2,4,6,8,9,13].

Histologically, MFB is characterized by well-circumscribed tumors composed of spindle cells arranged in short fascicles, interspersed with hyalinized collagen bands [1,2,5,8,9,13]. Various histological subtypes can add to the diagnostic complexity [1-4,6,8,9,13]. The epithelioid variant, for example, can mimic invasive lobular carcinoma, increasing the risk of misdiagnosis [3,4,6,8].

Diagnosis relies on tissue sampling, with core needle biopsy preferred over fine-needle aspiration, which may yield non-diagnostic samples [1,2,6,8,9,13]. Immunohistochemistry is pivotal in distinguishing MFB from other breast tumors [1,2,4,6,8,9,11,13]. MFB typically shows positive staining for vimentin and CD34, with variable positivity for desmin, smooth muscle actin (SMA), and hormone receptors (ER and PR) [1,3,4,6,7,8,9,11,13]. Notably, it is negative for cytokeratins, S100, and p63 [1,3-6,7,9-11,13]. Loss of Rb protein expression is another characteristic finding [2,3,11].

Due to the combined effect of non-specific imaging findings, the potential for misinterpretation on biopsy warrants local surgical excision with clear margins as the primary treatment of choice [1-3,5,6,8,9,11,13]. Additional treatments, such as radiation or systemic therapy, are not indicated [2,6]. The tumor’s benign nature and the absence of reported malignant transformation underscore the importance of avoiding overtreatment [1-6,7-9,11,13].

The prognosis for MFB is excellent [2,6,10]. Recurrence is rare following complete excision, and there are no documented cases of malignant transformation [1,2,5,6,9,10,11,13]. Follow-up care is generally focused on monitoring for local recurrence and ensuring surgical site healing, rather than disease progression [2,5,6,9,13].

In clinical practice, distinguishing MFB from other benign and malignant breast tumors requires a multidisciplinary approach [6]. Collaboration among radiologists, pathologists, and surgeons ensures accurate diagnosis and appropriate treatment [6]. Awareness of MFB’s varied presentations and histological subtypes is essential to avoid misdiagnosis and overtreatment [6].

## Conclusions

This case of myofibroblastoma of the breast, though rare, is an important consideration in the differential diagnosis of breast lesions due to its varied presentations and histological subtypes. Accurate diagnosis is essential to avoid misdiagnosis and overtreatment, relying on a combination of clinical evaluation, imaging, and core needle biopsy with immunohistochemistry. In summary, the combined effect of non-specific imaging findings, the potential for misinterpretation on biopsy and the need to distinguish it from other spindle cell lesions demonstrates the need for complete surgical excision. Long-term follow-up, while rarely needed, can monitor for recurrence.

This case highlights the significance of recognizing MFB’s diverse patterns to ensure appropriate management. Further research is needed to deepen understanding of its etiology and pathogenesis. Increasing awareness among clinicians will lead to timely, accurate diagnosis and optimal patient outcomes.
